# Epidemiology and healthcare burden of implant-associated complications in Germany: a nationwide population-based analysis

**DOI:** 10.1007/s00068-026-03292-4

**Published:** 2026-08-24

**Authors:** Houmam Anees, Christian Heiss, Thaqif El Khassawna

**Affiliations:** 1 Department of Trauma, Hand and Reconstructive Surgery, Faculty of Medicine, Justus-Liebig-University of Giessen, 35392 Giessen, Germany; 2 Experimental Trauma Surgery, Faculty of Medicine, Justus-Liebig-University of Giessen, 35392 Giessen, Germany; 3 Biruni University, Istanbul, Türkiye; 4 School of Pharmacy, The University of Jordan, Amman, 11942 Jordan

**Keywords:** Implant-associated complications, Trauma surgery, Revision surgery, Periprosthetic joint infection, Diagnosis-related groups, Healthcare costs

## Abstract

**Purpose:**

Implant-associated complications remain a major challenge in orthopaedic and trauma surgery, particularly among elderly patients requiring revision procedures or infection management. This study analysed nationwide trends and healthcare burden of implant-associated complications in Germany.

**Methods:**

Nationwide German hospital discharge data from 2000 to 2023 were analysed and complemented by Diagnosis-Related Group (DRG) data from 2024. Cases were identified using ICD-10-GM code T84 and its subcategories. Outcomes included temporal trends, age-standardised incidence, demographic characteristics, in-hospital mortality, length of stay (LOS), and DRG-based healthcare resource utilisation.

**Results:**

Hospitalisations for implant-associated complications increased from 45,121 in 2000 to a peak of 95,154 in 2011, followed by a decline to 72,846 in 2023. Age-standardised incidence decreased by approximately 31% between 2010 and 2023. Segmented regression identified a temporal breakpoint in 2010.6, with annual percentage changes of + 6.21% before and − 3.55% after the breakpoint. Annual hospitalisations for periprosthetic joint infection increased from 5,367 to 11,215, while osteosynthesis-associated infections increased from 2,469 to 7,059. Mean LOS decreased from 20.8 to 11.8 days, whereas in-hospital mortality remained stable. Compared with low-complexity cases, infection-related complications were associated with prolonged hospitalisation and approximately threefold higher treatment costs, while complex revision procedures generated mean costs exceeding €9,000 per case.

**Conclusion:**

Although the age-standardised incidence of implant-associated complications has declined in recent years, infection-related complications and complex revision procedures continue to impose a substantial clinical and economic burden, underlining the need for continued optimisation of prevention and treatment strategies.

**Supplementary Information:**

The online version contains supplementary material available at 10.1007/s00068-026-03292-4.

## Highlights

Segmented regression identified a temporal trend change around 2010–2011, followed by a sustained decline in age-standardised incidence.

Patients were predominantly elderly, with more than two-thirds aged ≥ 65 years.

Mean length of stay declined substantially over time, while in-hospital mortality remained relatively stable.

Infection-related complications and revision procedures were associated with prolonged hospitalisation and substantially higher treatment costs.

Despite declining incidence, implant-associated infections continue to impose a considerable clinical and economic burden.

**Supplementary Information:**

The online version contains supplementary material available at 10.1007/s00068-026-03292-4.

## Introduction

Implant-related surgical procedures, including joint arthroplasty and fracture fixation using osteosynthesis devices, represent highly effective interventions that substantially improve pain, mobility, and quality of life [1]. Owing to demographic changes, increasing life expectancy, and expanding indications, the number of primary joint arthroplasties has steadily increased over recent decades and is expected to continue rising in the future [2]. Projections from the United States estimate substantial increases in arthroplasty demand by 2030, including growth of 174% for total hip arthroplasties and up to 673% for total knee arthroplasties [3]. Consequently, the number of revision procedures is also expected to rise considerably, further increasing the clinical and economic burden on healthcare systems [3].

Among implant-associated complications, periprosthetic joint infection (PJI) is considered one of the most severe and clinically challenging conditions. PJI is associated with substantial morbidity and may result in prolonged treatment courses, reinfection, disarticulation of the affected limb, and even death [4]. In a nationwide analysis including 52,286 patients treated for PJI in Germany, an in-hospital mortality rate of 3.5% was reported, highlighting the clinical severity of this complication [4]. Severe complications such as systemic inflammatory response syndrome (SIRS), septic shock, and organ failure were identified as major contributors to mortality [4, 5].

In addition to its clinical impact, PJI is associated with substantial healthcare utilisation. Patients frequently require prolonged hospitalisation, with a mean length of stay of 20.4 days, and approximately 25% of cases necessitate intensive care treatment [4]. These findings underscore the considerable resource demands associated with managing implant-associated infections. As the number of implanted orthopaedic devices continues to increase, the absolute number of implant-associated complications is also expected to rise.

In trauma and reconstructive surgery, implant-related complications predominantly affect elderly and multimorbid patients, a population that is rapidly expanding due to demographic changes. Epidemiological projections indicate a substantial increase in arthroplasty procedures driven primarily by population ageing rather than changes in incidence rates [6].

Implant-associated complications comprise a broad spectrum of adverse events extending beyond infection alone. According to the ICD-10-GM classification, T84-coded complications include mechanical complications, loosening, implant failure, dislocation, osteosynthesis-related complications, and infection-associated conditions [7]. Collectively, these complications represent a major cause of hospitalisation, revision surgery, prolonged treatment, and resource demands in orthopaedic and trauma surgery.

While previous studies have primarily focused on isolated aspects such as periprosthetic joint infection or revision arthroplasty, comprehensive nationwide analyses integrating long-term epidemiological trends, demographic characteristics, clinical severity, and healthcare burden of implant-associated complications remain scarce. In particular, population-based investigations evaluating implant-associated complications across both elective arthroplasty and trauma-related settings within a unified framework are lacking.

Therefore, this study aimed to analyse nationwide trends in implant-associated complications in Germany over more than two decades and to characterise their epidemiology, mortality, and healthcare burden. By combining population-based epidemiological data with diagnosis-related group (DRG)-derived analyses, this study provides a comprehensive assessment of the burden of implant-associated complications and their implications for modern orthopaedic and trauma care.

## Methods

### Study design and data source

This retrospective population-based study was conducted using nationwide hospital discharge data from Germany between 2000 and 2023 [8], complemented by Diagnosis Related Group (DRG) data from 2024 provided by the Institut für das Entgeltsystem im Krankenhaus (InEK) [9]. The study aimed to assess temporal trends, demographic characteristics, clinical severity, and healthcare resource utilisation of implant-associated complications.

Age-standardised incidence rates were obtained directly from the German Federal Health Monitoring (GBE) database, where they are reported based on the German standard population (2022) [8]. The GBE database provides data for the years 2000, 2005, 2010, and annually from 2011 onwards; therefore, intermediate years were not available for analysis.

While the GBE database was used to assess long-term epidemiological trends, the 2024 Diagnosis-Related Group (DRG) dataset provided by the Institut für das Entgeltsystem im Krankenhaus (InEK) was used exclusively to characterise contemporary healthcare resource utilisation and the economic burden associated with implant-related complications [9]. Accordingly, temporal trend analyses were based solely on the nationwide hospital discharge data from 2000 to 2023.

### Case definition

Cases were identified using the International Classification of Diseases, 10th Revision (ICD-10-GM) code T84, including all subcategories representing complications related to orthopaedic implants, endoprostheses, and osteosynthesis devices. These include both mechanical complications and infection-related conditions. All inpatient hospitalisations with a primary or secondary diagnosis of T84 were included. The analysis was based on hospital discharge data and therefore reflects hospitalisations (hospital cases), rather than individual patients.

### Variables and outcomes

The primary outcome was the temporal trend of implant-associated complications, assessed by annual case numbers and age-standardised incidence rates.

Secondary outcomes included demographic characteristics (age and sex distribution), in-hospital mortality, healthcare utilisation measures (length of stay [LOS] and proportion of short-stay hospitalisations), and economic parameters derived from Diagnosis Related Group (DRG) data, including mean treatment costs and healthcare resource utilisation.

To illustrate differences in healthcare resource utilisation across varying levels of clinical complexity, illustrative DRGs were selected based on their clinical relevance and frequency among implant-associated complications. DRG I71B was chosen to represent relatively low-complexity cases, DRG I12C to represent infection-related conditions, and DRG I47A to represent revision procedures associated with high resource utilisation.

### Statistical analysis

Descriptive statistics were used to summarise all variables. Continuous variables are presented as means, while categorical variables are reported as absolute numbers and percentages. No imputation of missing data was performed.

To further evaluate temporal trends, segmented regression analysis was performed using the available observation years. Age-standardised incidence rates were log-transformed and modelled as a function of calendar year. The model was used to estimate the temporal breakpoint and corresponding annual percentage changes (APCs) before and after the identified breakpoint, with 95% confidence intervals. Analyses were performed using R version 4.6.1 and the segmented package for R [10].

## Ethical considerations

This study was based on anonymised, aggregated administrative data and did not involve direct patient contact or identifiable information. According to national regulations, formal ethical approval was not required.

## Results

### Temporal trends in implant-associated complications

**Fig. 1:**
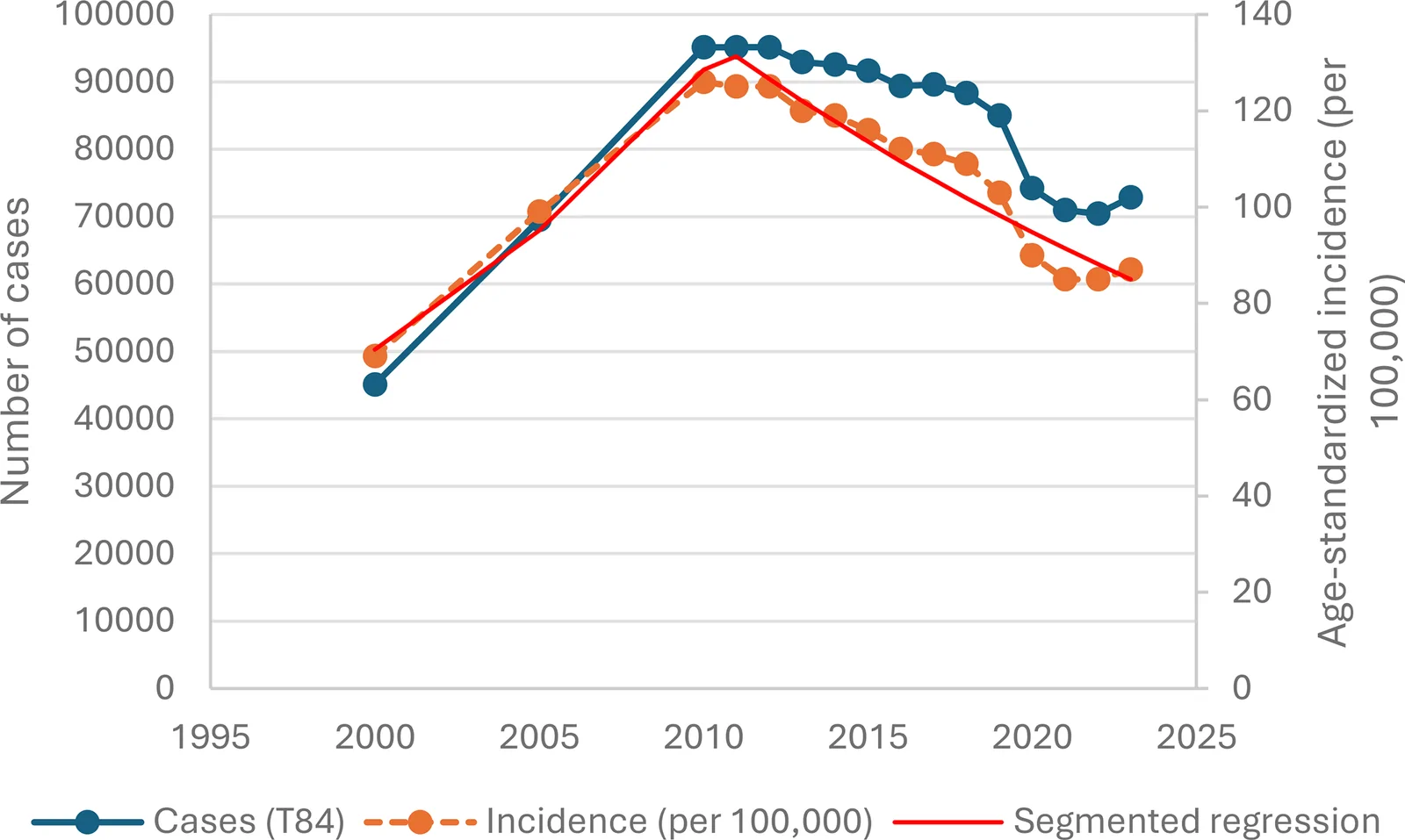
Nationwide trends in implant-associated complications in Germany between 2000 and 2023. The solid blue line represents annual hospitalisations (left axis), whereas the dashed orange line shows age-standardised incidence rates per 100,000 population (right axis). The solid red line represents the segmented regression model fitted to the observed age-standardised incidence rates

Between 2000 and 2023, the annual number of inpatient hospitalisations for implant-associated complications (ICD-10-GM T84) in Germany increased markedly, rising from 45,121 cases in 2000 to a peak of 95,154 cases in 2011. Thereafter, a gradual decline was observed, reaching 72,846 cases in 2023. A similar pattern was observed for age-standardised incidence rates, which increased until approximately 2010–2011 and subsequently declined over the following decade (Fig. 1).

The most pronounced decrease occurred during the COVID-19 pandemic period, with case numbers dropping from 84,981 in 2019 to 74,254 in 2020. Although a partial recovery was observed thereafter, case numbers remained below pre-pandemic levels in 2023.

Segmented regression analysis of age-standardised incidence rates identified a temporal breakpoint in 2010.6 (95% CI 2009.4–2011.8). Before this breakpoint, the annual percentage change (APC) was + 6.21% (95% CI 4.71–7.72%; *p* < 0.001), whereas after the breakpoint, the APC was − 3.55% (95% CI − 4.27 to − 2.84%; *p* < 0.001).

### Subgroup analysis of infection-related complications

**Fig. 2:**
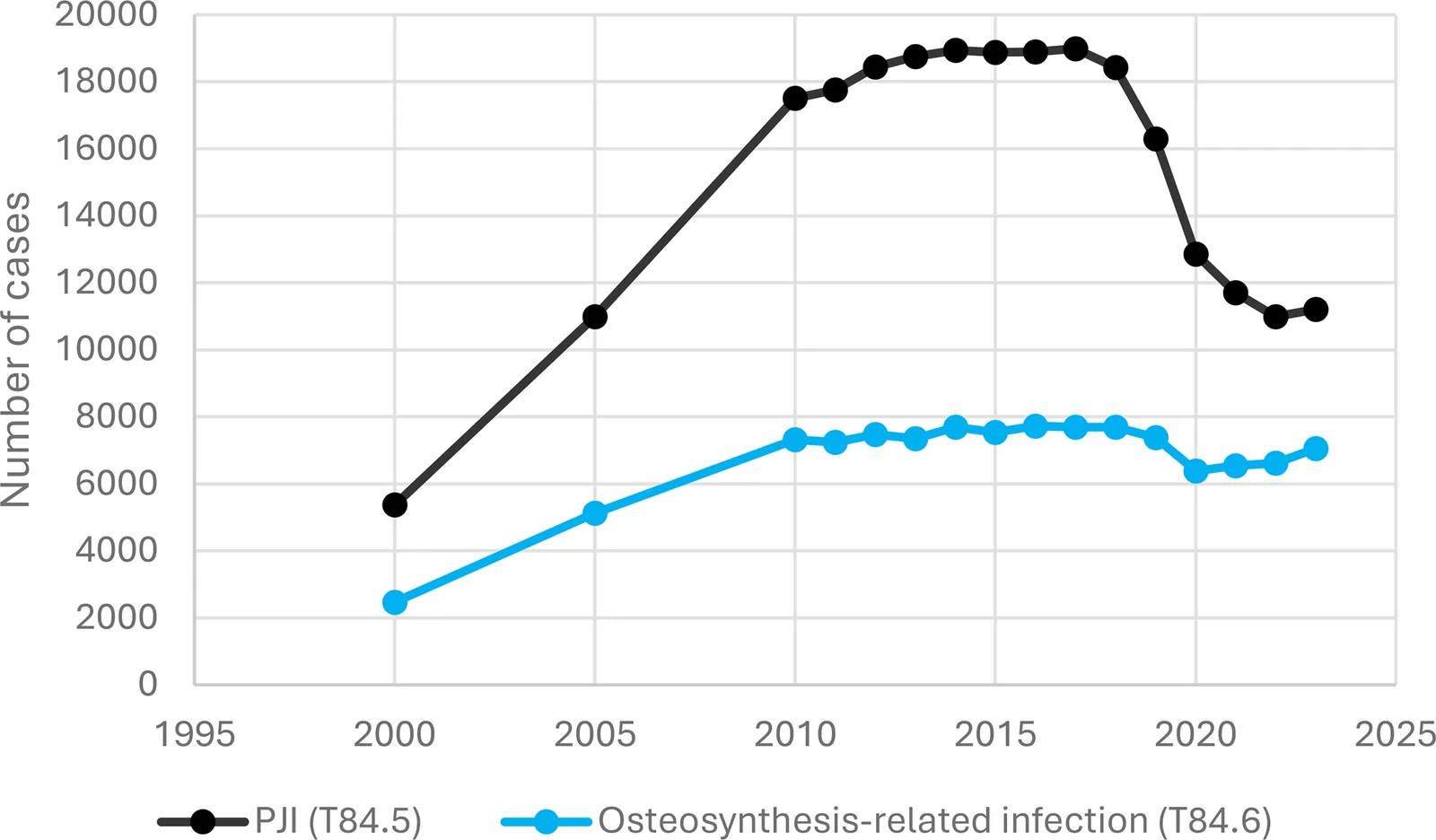
Trends in infection-related implant complications in Germany from 2000 to 2023. The solid lines represent infections associated with joint prostheses (T84.5) and internal fixation devices (T84.6)

Subgroup analysis demonstrated that infection-related complications constituted a substantial proportion of all implant-associated complications. Cases of periprosthetic joint infection (PJI; T84.5) increased steadily from 5,367 cases in 2000 to a peak of approximately 18,900 cases between 2014 and 2017, followed by a marked decline to 11,215 cases in 2023. A comparable trend was observed for osteosynthesis-related infections (T84.6), which increased from 2,469 cases in 2000 to approximately 7,700 cases in the mid-2010s before declining to 7,059 cases in 2023 (Fig. 2).

Despite the overall decline in recent years, both subgroups remained at substantially higher levels than in the early observation period, indicating a persistent burden of infection-related complications. The distribution of all ICD-10-GM T84 subcategories across selected years is provided in Supplementary Table S2. Mechanical complications of joint prostheses (T84.0) consistently represented the largest subgroup, followed by periprosthetic joint infections (T84.5) and infection-related complications of internal fixation devices (T84.6).

### Demographic characteristics, healthcare utilisation, and mortality

Across the study period, implant-associated complications occurred more frequently in female patients, accounting for approximately 57–59% of cases, while male patients represented 41–43%. The majority of cases were observed in older age groups, particularly among patients aged ≥ 65 years.

Healthcare utilisation patterns changed substantially over time. Mean length of stay (LOS) decreased continuously from 20.8 days in 2000 to 11.8 days in 2023. In parallel, the proportion of short-stay hospitalisations (1–3 days) increased from 9.4% to 22.4%, indicating a shift towards shorter inpatient treatment durations. In-hospital mortality rates remained relatively stable throughout the observation period, increasing slightly from 1.03% in 2010 to 1.34% in 2023 despite declining case numbers (Table 1).

**Table 1:** Overview of case volume, demographic characteristics, healthcare utilisation, and in-hospital mortality of implant-associated complications (ICD-10-GM T84) in Germany across selected years. Variables include total case numbers, sex distribution, mean length of stay (LOS), proportion of short-stay hospitalisations, and in-hospital mortality rates

Year	Total cases	Male (%)	Female (%)	Mean LOS (days)	Short stay (%)	In-hospital mortality (%)
2010	95,094	41.0	59.0	15.5	13.6	**1.03**
2015	91,645	42.3	57.7	14.3	17.6	**1.22**
2020	74,254	42.7	57.3	12.3	21.6	**1.33**
2023	72,846	42.5	57.5	11.8	22.4	**1.34**

LOS: length of stay. Short stay was defined as a hospital stay of 1–3 days. In-hospital mortality is presented as the percentage of hospitalisations resulting in death. Data were derived from nationwide German hospital discharge statistics

A detailed overview of annual case numbers, demographic characteristics, healthcare utilisation measures, and mortality across the entire observation period is provided in Supplementary Table S1.

### Healthcare resource utilisation

Analysis of selected diagnosis-related groups (DRGs) revealed substantial differences in healthcare resource utilisation. Cases classified under DRG I71B (muscle and soft tissue disorders) were associated with relatively low resource consumption, with a mean LOS of 4.1 days and mean costs of approximately €1,954 per case. In contrast, infection-related cases (DRG I12C) demonstrated a markedly higher burden, with a mean LOS of 10.4 days and costs of approximately €6,060.

**Fig. 3:**
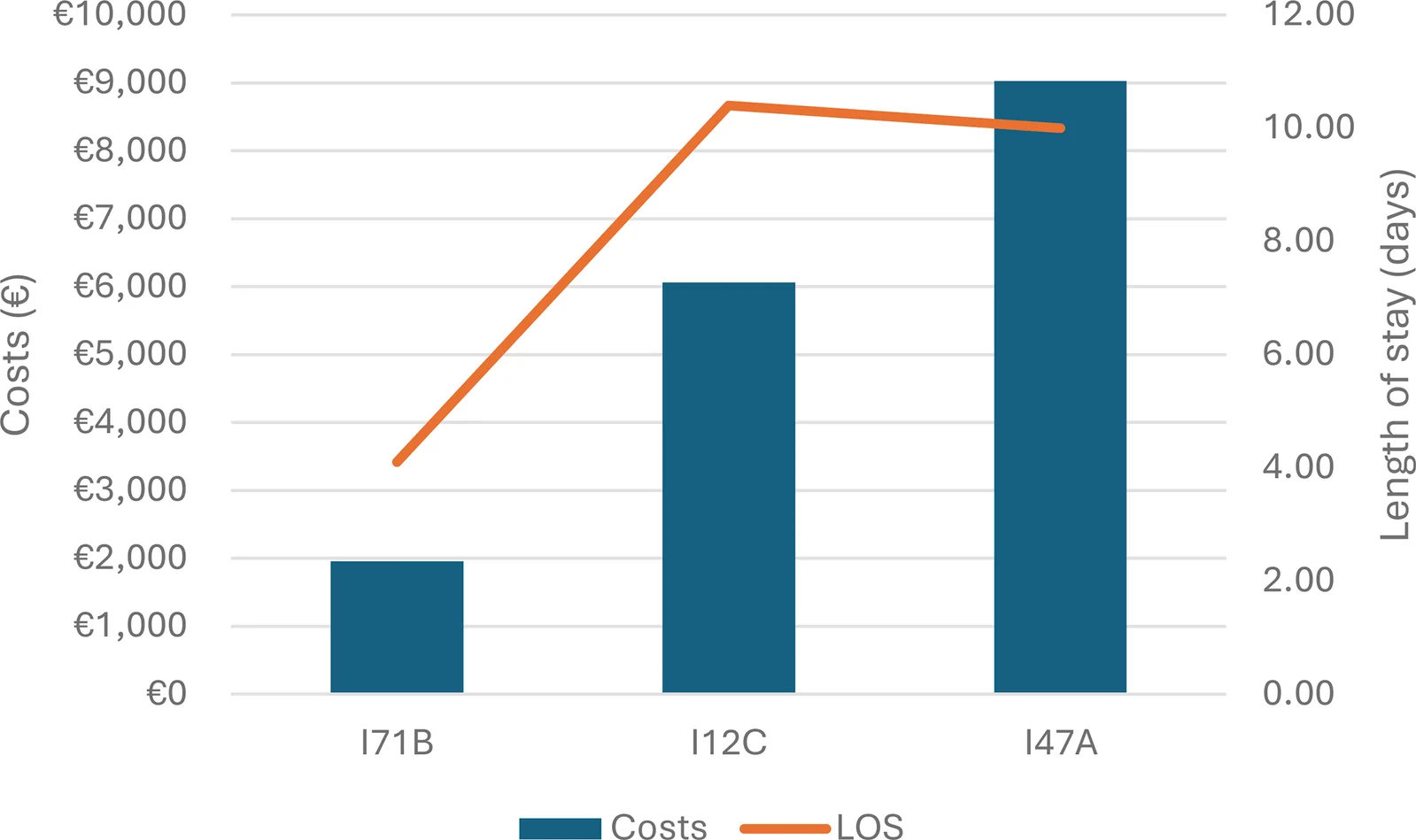
Comparison of healthcare resource utilisation across selected diagnosis-related groups (DRGs). Bars represent mean treatment costs per case (€), while the line indicates mean length of hospital stay (LOS) in days

The highest resource utilisation was observed in revision procedures (DRG I47A), which were associated with mean costs of approximately €9,024 per case and a mean LOS of 10.0 days. Overall, both LOS and treatment costs increased substantially with clinical complexity, particularly in infection-related and revision cases (Fig. 3).

The selected DRGs represent increasing levels of clinical complexity and resource utilisation, ranging from relatively uncomplicated conditions (I71B) to infection-related cases (I12C) and complex revision procedures (I47A).

## Discussion

### Principal findings

The present nationwide analysis demonstrates that implant-associated complications in Germany have undergone a characteristic temporal evolution over the past two decades. Following a marked increase until approximately 2010–2011, both absolute case numbers and age-standardised incidence rates declined steadily thereafter. Despite this overall decrease, infection-related complications, particularly periprosthetic joint infections (PJI) and osteosynthesis-associated infections, remained at substantially elevated levels compared to early observation periods, indicating a persistent and clinically relevant burden.

In parallel, healthcare utilisation patterns changed substantially, reflected by a continuous reduction in length of hospital stay and an increase in short-stay hospitalisations. At the same time, infection-related complications continued to impose a considerable burden on healthcare resources. These findings are consistent with previous studies demonstrating the substantial clinical and economic burden associated with implant-related infections and revision procedures [11, 12].

### Interpretation of temporal trends

The segmented regression analysis identified a temporal breakpoint around 2010–2011, corresponding to the period during which age-standardised incidence reached its maximum. This finding is consistent with the descriptive trends observed in the nationwide data and suggests a transition from a prolonged phase of increasing implant-associated complications to a sustained decline thereafter rather than an abrupt change attributable to a single event.

The observed increase until the early 2010s likely reflects the substantial expansion of orthopaedic and trauma procedures during the preceding decade, particularly the rising number of joint arthroplasties and osteosynthesis interventions. Previous registry-based analyses demonstrated a continuous increase in arthroplasty and revision procedure volumes worldwide, with infection-related complications representing an increasingly important indication for revision surgery [11].

The subsequent decline was likely multifactorial and may reflect improvements in perioperative care, broader implementation of infection prevention measures, optimisation of surgical techniques, and changes in clinical practice. However, owing to the descriptive nature of the present study and the use of aggregated administrative data, the specific factors underlying the observed temporal transition cannot be determined.

The pronounced decrease observed during the COVID-19 pandemic is consistent with the widespread disruption of surgical services during this period. Although infection-related complications frequently require urgent surgical treatment, reduced elective implantation volumes, altered referral patterns, postponement of selected revision procedures, and changes in healthcare utilisation may all have contributed to the observed decline. The segmented regression model was designed to identify the principal long-term temporal trend and therefore did not specifically account for this short-term disruption. Because only four post-pandemic observation years (2020–2023) were available, modelling an additional COVID-19-related breakpoint would not have provided a robust estimate. Consequently, the post-2011 APC should be interpreted as reflecting the overall long-term decline rather than the isolated effect of the pandemic. The incomplete recovery of case numbers by 2023 suggests that some of these changes may have persisted beyond the acute pandemic period.

### Burden of infection-related complications

A key finding of this study is the sustained high burden of infection-related implant complications. Both periprosthetic joint infections (PJI) and osteosynthesis-associated infections showed a steep increase until the mid-2010s, followed by a decline that nevertheless remained substantially above baseline levels observed during the early observation period. These findings indicate that, despite advances in surgical care, infection-related complications continue to represent a substantial clinical challenge owing to their complex management and adverse clinical consequences [13, 14].

The persistence of high case numbers may reflect the increasing complexity of treated patients, particularly elderly and multimorbid individuals. Previous nationwide analyses demonstrated a continuous increase in fracture-related infections in Germany, especially among older patients [15]. In addition, bacterial biofilm formation on implant surfaces contributes substantially to treatment difficulty and chronic infection persistence [16].

An additional noteworthy finding was the predominance of female patients among hospitalisations for implant-associated complications throughout the study period. As the present analysis did not include data on the underlying number of orthopaedic implant procedures by sex, it remains uncertain whether this observation reflects differences in the distribution of implant procedures or sex-specific differences in the occurrence of implant-associated complications.

### Healthcare resource utilisation

Our DRG-based analysis demonstrated marked differences in healthcare resource utilisation according to clinical complexity. Compared with less complex cases (DRG I71B), infection-related cases (DRG I12C) and revision procedures (DRG I47A) were associated with substantially higher treatment costs and longer hospital stays, confirming the considerable economic burden of implant-associated complications. These findings are consistent with previous reports showing that implant-associated infections frequently require prolonged hospitalisation, repeated surgical interventions, long-term antimicrobial therapy, intensive rehabilitation, and complex multidisciplinary care pathways [12, 17].

Several studies have demonstrated that revision arthroplasty procedures are significantly more expensive than primary arthroplasty. In a recent analysis of revision total hip arthroplasty (rTHA), the median cost per episode of care was 76% higher than primary THA, with prosthetic joint infection representing the most expensive indication for revision surgery [17]. The main cost drivers included prolonged ward-related costs, surgical theatre expenses, implant costs, and intensive care utilisation. Patients undergoing two-stage revision procedures for PJI incurred particularly high cumulative treatment costs due to multiple admissions and repeated operations [17].

Similarly, fracture-related infections substantially increase healthcare expenditures compared to uncomplicated fracture treatment. A German Level 1 trauma centre study demonstrated that FRI treatment costs were between 1.8-fold and 4.7-fold higher than initial fracture management depending on fracture localisation [12]. Increased costs were primarily associated with repeated surgeries, prolonged hospital stays, and additional inpatient care requirements.

These findings are consistent with previous studies demonstrating that implant-associated infections are associated with increased morbidity, higher reoperation rates, prolonged hospitalisation, and substantially increased healthcare costs compared with non-infected patients [13].

Our findings further support the considerable healthcare burden associated with musculoskeletal infections. Nationwide German data demonstrated increasing rates of fracture-related infections over the last decade, particularly among elderly patients, highlighting the anticipated future strain on healthcare resources [15].

### Strengths and limitations

A major strength of this study is the use of nationwide inpatient data derived from the German hospital discharge statistics, enabling a comprehensive and population-based assessment of implant-associated complications across the entire healthcare system. The large sample size and long observation period from 2000 to 2023 allow robust evaluation of epidemiological trends, including the impact of major healthcare disruptions such as the COVID-19 pandemic. In addition, the standardised ICD-10-GM coding framework ensures a high level of consistency in case identification and comparability over time.

Several limitations must nevertheless be acknowledged. First, the analysis is based on administrative data and may therefore be subject to coding inaccuracies or changes in coding practices over time. Second, the dataset is restricted to inpatient hospitalisations and does not capture outpatient management of implant-associated complications, potentially underestimating the overall burden. Third, the aggregated nature of the data precludes patient-level analyses and does not allow adjustment for relevant clinical variables such as comorbidities, implant type, surgical technique, microbiological findings, or infection severity. In addition, because data on the total number of orthopaedic implant procedures were not available within the analysed datasets, complication rates relative to implantation volume could not be determined. Furthermore, the publicly available dataset does not distinguish between primary and secondary T84 diagnoses, precluding subgroup analyses according to diagnosis position. Fourth, potential shifts in clinical practice patterns, including earlier interventions or changes in admission thresholds, cannot be fully assessed within this framework. Finally, due to the descriptive study design, causal inferences regarding the underlying drivers of observed trends cannot be established.

## Conclusions

Implant-associated complications in Germany demonstrated a marked decline in recent years following a peak in the early 2010s. Nevertheless, infection-related complications remain a substantial and persistent clinical and economic burden. Although hospital stays became progressively shorter over the study period, complex cases, particularly implant-associated infections and revision procedures, continue to require extensive healthcare resources and multidisciplinary management.

Given ongoing demographic ageing and the biological complexity of implant-associated infections, continued optimisation of preventive strategies, perioperative management, and infection treatment pathways remains essential to reduce the future burden of implant-associated complications in orthopaedic and trauma surgery.

## Supplementary Information

Below is the link to the electronic supplementary material.

Supplementary Material 1

Supplementary Material 2

## Data Availability

The datasets analysed during the current study are publicly available from the German Federal Health Monitoring (GBE) database and the Institute for the Hospital Remuneration System (InEK).

## References

[1] Rupp M, Walter N, Lau E, Worlicek M, Kurtz SM, Alt V. Recent trends in revision knee arthroplasty in Germany. Sci Rep. 2021;11. 10.1038/s41598-021-94988-7. 10.1038/s41598-021-94988-7 PMC8322047 34326421

[2] Worlicek M, Koch M, Daniel P, Freigang V, Angele P, Alt V, Kerschbaum M, Rupp M. A retrospective analysis of trends in primary knee arthroplasty in Germany from 2008 to 2018. Sci Rep. 2021;11. 10.1038/s41598-021-84710-y. 10.1038/s41598-021-84710-y PMC7933436 33664448

[3] Kurtz S, Ong K, Lau E, Mowat F, Halpern M. Projections of Primary and Revision Hip and Knee Arthroplasty in the United States from 2005 to 2030. J Bone Joint Surg. 2007;89:780–5. 10.2106/JBJS.F.00222. 17403800 10.2106/JBJS.F.00222

[4] Reinhard J, Lang S, Walter N, Schindler M, Bärtl S, Szymski D, Alt V, Rupp M. In-hospital mortality of patients with periprosthetic joint infection demographic, comorbidity, and complication profiles of 52,286 patients, (n.d.). 10.1302/2633-1462 10.1302/2633-1462.54.BJO-2023-0162.R1 PMC11045279 38663864

[5] Xu Y, Huang TB, Schuetz MA, Choong PFM. Mortality, patient-reported outcome measures, and the health economic burden of prosthetic joint infection. EFORT Open Rev. 2023;8:690–7. 10.1530/EOR-23-0078. 37655835 10.1530/EOR-23-0078 PMC10548306

[6] Pilz V, Hanstein T, Skripitz R. Projections of primary hip arthroplasty in Germany until 2040. Acta Orthop. 2018;89:308–13. 10.1080/17453674.2018.1446463. 29504824 10.1080/17453674.2018.1446463 PMC6055773

[7] Bundesinstitut für Arzneimittel und Medizinprodukte (BfArM). ICD-10-GM 2023: T84 Complications of internal orthopaedic prosthetic devices, implants and grafts., (n.d.). https://klassifikationen.bfarm.de/icd-10-gm/kode-suche/htmlgm2023/block-t80-t88.htm. Accessed June 22, 2026.

[8] Gesundheitsberichterstattung DESBUNDES, editor. (n.d.). https://www.gbe-bund.de/gbe/. Accessed December 17, 2025.

[9] Institut für das Entgeltsystem im Krankenhaus (InEK), (n.d.). https://datenbrowser.inek.org. Accessed March 12, 2026.

[10] Muggeo VMR. Segmented: an R package to fit regression models with broken-line relationships. 2008. https://journal.r-project.org/articles/RN-2008-004/RN-2008-004.pdf. Accessed June 28, 2026.

[11] Sadoghi P, Koutp A, Prieto DP, Clauss M, Kayaalp ME, Hirschmann MT. The projected economic burden and complications of revision hip and knee arthroplasties: Insights from national registry studies, Knee Surgery, Sports Traumatology. Arthroscopy. 2025;33:3211–7. 10.1002/ksa.12678. 10.1002/ksa.12678 PMC12392383 40221912

[12] Walter N, Neubauer I, Baertl S, Alt V, Rupp M. Direct healthcare cost of fracture-related infection treatment. JBJS Open Access. 2025;10. 10.2106/JBJS.OA.24.00133. 10.2106/JBJS.OA.24.00133 PMC12367026 40851846

[13] Badia JM, Casey AL, Petrosillo N, Hudson PM, Mitchell SA, Crosby C. Impact of surgical site infection on healthcare costs and patient outcomes: a systematic review in six European countries. J Hosp Infect. 2017;96:1–15. 10.1016/j.jhin.2017.03.004. 28410761 10.1016/j.jhin.2017.03.004

[14] Widmer A, Imhasly N, Brand C, Zdravkovi V, Spoerri A, Schmidlin K, Wicki M, Beck M, Sommerstein R. Increased long-term mortality of patients with prosthetic joint infection after primary total hip arthroplasty – A large observational cohort study. J Infect. 2026;92:106689. 10.1016/j.jinf.2026.106689. 41534600 10.1016/j.jinf.2026.106689

[15] Walter N, Rupp M, Lang S, Alt V. The epidemiology of fracture-related infections in Germany. Sci Rep. 2021;11. 10.1038/s41598-021-90008-w. 10.1038/s41598-021-90008-w PMC8128870 34001973

[16] Kalali N, Ebrahimzadeh MH, Moradi A, Jirofti N. Periprosthetic Joint Infection (PJI). Archives Bone Joint Surg. 2025;13:406–13. 10.22038/abjs.2025.81816.3726. 10.22038/ABJS.2025.81816.3726 PMC12335196 40787294

[17] Hammat AS, D’Apollonio T, Nelson R, Campbell D, Ramasamy B, Solomon LB, Gnanamanickam ES, Callary SA. The Higher Hospital Costs of Revision Total Hip Arthroplasty by Diagnosis: Analysis of Cost Drivers and Periprosthetic Joint Infection Treatment Pathways. ANZ J Surg. 2025. 10.1111/ans.70368. 41199612 10.1111/ans.70368

