# Supplementary material for: Epidemiology and healthcare burden of implant-associated complications in Germany: a nationwide population-based analysis
Source: Eur J Trauma Emerg Surg. 2026 Aug 24;52(1):259. doi: 10.1007/s00068-026-03292-4 (PMC13503490; doi:10.1007/s00068-026-03292-4)
Supplement: Supplementary file 1 [file 68_2026_3292_MOESM1_ESM.docx]

**Epidemiology and healthcare burden of implant-associated complications in Germany: a nationwide population-based analysis**

Houmam Anees^1,2^*****, Christian Heiß^1,2,3^ and Thaqif El Khassawna^2,4^

^1^Department of Trauma, Hand and Reconstructive Surgery, Faculty of Medicine, Justus-Liebig-University of Giessen, 35392 Giessen, Germany

^2^Experimental Trauma Surgery, Faculty of Medicine, Justus-Liebig-University of Giessen, 35392 Giessen, Germany

^3^Biruni University, Istanbul, Türkiye

^4^School of Pharmacy, The University of Jordan, Amman 11942, Jordan

***Corresponding Author:** [Houmam.Anees@chiru.med.uni-giessen.de](mailto:Houmam.Anees@chiru.med.uni-giessen.de) (H. Anees).

| **Year** | **Cases** | **LOS** | **Mortality** | **Short stay (%)** |
| --- | --- | --- | --- | --- |
| 2000 | 45,121 | 20.8 | 333 | 9.4 |
| 2005 | 69,571 | 17.4 | 739 | 10.4 |
| 2010 | 95,094 | 15.5 | 977 | 13.6 |
| 2011 | 95,154 | 15.2 | 911 | 14.0 |
| 2012 | 95,154 | 14.8 | 922 | 14.4 |
| 2013 | 92,932 | 14.8 | 1,053 | 15.2 |
| 2014 | 92,586 | 14.5 | 1,003 | 16.8 |
| 2015 | 91,645 | 14.3 | 1,118 | 17.6 |
| 2016 | 89,392 | 14.2 | 1,048 | 17.8 |
| 2017 | 89,578 | 13.9 | 1,159 | 18.7 |
| 2018 | 88,332 | 13.8 | 1,134 | 18.9 |
| 2019 | 84,981 | 13.3 | 1,088 | 19.7 |
| 2020 | 74,254 | 12.3 | 985 | 21.6 |
| 2021 | 70,980 | 12.1 | 986 | 22.0 |
| 2022 | 70,410 | 11.9 | 1,018 | 22.8 |
| 2023 | 72,846 | 11.8 | 979 | 22.4 |

**Supplementary Table S1:** Nationwide annual number of inpatient hospitalisations for implant-associated complications (ICD-10-GM T84) in Germany and corresponding clinical characteristics from 2000 to 2023. Variables include total case numbers, mean length of hospital stay (LOS), in-hospital mortality, and proportion of short-stay hospitalisations. The primary analysis focuses on the years 2010–2023, for which complete annual nationwide data were available. Data for the years 2000 and 2005 are presented as historical reference points.

LOS: length of stay. Short stay defined as hospital stay of 1–3 days. Data derived from nationwide hospital discharge statistics.
