# Supplementary material for: Epidemiology and healthcare burden of implant-associated complications in Germany: a nationwide population-based analysis
Source: Eur J Trauma Emerg Surg. 2026 Aug 24;52(1):259. doi: 10.1007/s00068-026-03292-4 (PMC13503490; doi:10.1007/s00068-026-03292-4)
Supplement: Supplementary file 2 [file 68_2026_3292_MOESM2_ESM.docx]

**Epidemiology and healthcare burden of implant-associated complications in Germany: a nationwide population-based analysis**

Houmam Anees^1,2^*****, Christian Heiß^1,2,3^ and Thaqif El Khassawna^2,4^

^1^Department of Trauma, Hand and Reconstructive Surgery, Faculty of Medicine, Justus-Liebig-University of Giessen, 35392 Giessen, Germany

^2^Experimental Trauma Surgery, Faculty of Medicine, Justus-Liebig-University of Giessen, 35392 Giessen, Germany

^3^Biruni University, Istanbul, Türkiye

^4^School of Pharmacy, The University of Jordan, Amman 11942, Jordan

***Corresponding Author:** [Houmam.Anees@chiru.med.uni-giessen.de](mailto:Houmam.Anees@chiru.med.uni-giessen.de) (H. Anees).

| **ICD-10-GM subcategory** | **2000** | **2005** | **2010** | **2015** | **2020** | **2023** |
| --- | --- | --- | --- | --- | --- | --- |
| T84.0 Mechanical complication of internal joint prosthesis | 28,313 | 38,898 | 47,444 | 41,883 | 34,732 | 34,905 |
| T84.1 Mechanical complication of internal fixation device of limb bones | 1,836 | 5,926 | 10,306 | 10,273 | 10,394 | 10,132 |
| T84.2 Mechanical complication of internal fixation device of other bones | 947 | 2,658 | 4,670 | 5,951 | 5,347 | 5,801 |
| T84.3 Mechanical complication of other bone devices, implants or grafts | 313 | 440 | 569 | 507 | 267 | 236 |
| T84.4 Mechanical complication of other internal orthopaedic devices, implants or grafts | 1,428 | 1,730 | 2,105 | 2,248 | 1,646 | 1,439 |
| T84.5 Infection and inflammatory reaction due to internal joint prosthesis | 5,367 | 10,987 | 17,515 | 18,945 | 12,857 | 11,215 |
| T84.6 Infection and inflammatory reaction due to internal fixation device | 2,469 | 5,123 | 7,317 | 7,692 | 6,380 | 7,059 |
| T84.7 Infection and inflammatory reaction due to other orthopaedic devices, implants or grafts | 581 | 841 | 1,052 | 825 | 538 | 538 |
| T84.8 Other complications of orthopaedic prosthetic devices, implants or grafts | 1,712 | 2,450 | 3,753 | 3,131 | 1,786 | 1,325 |
| T84.9 Unspecified complication of orthopaedic prosthetic devices, implants or grafts | 2,155 | 518 | 363 | 190 | 307 | 196 |

**Supplementary Table S2:** Distribution of inpatient hospitalisations across ICD-10-GM T84 subcategories in Germany during selected observation years. The table summarises the distribution of mechanical complications, infection-related complications, and other implant-associated disorders. To improve readability, selected reference years (2000, 2005, 2010, 2015, 2020, and 2023) are presented. Data were derived from the German Federal Health Monitoring (GBE) database. ICD-10-GM = German Modification of the International Classification of Diseases, 10th Revision.
